# Effect of Pleistocene Climatic Oscillations on the Phylogeography and Demography of Red Knobby Newt (*Tylototriton shanjing*) from Southwestern China

**DOI:** 10.1371/journal.pone.0056066

**Published:** 2013-02-12

**Authors:** Guohua Yu, Mingwang Zhang, Dingqi Rao, Junxing Yang

**Affiliations:** 1 State Key Laboratory of Genetic Resources and Evolution, Kunming Institute of Zoology, Chinese Academy of Sciences, Kunming, Yunnan, China; 2 University of Chinese Academy of Sciences, Beijing, China; 3 College of Animal Science and Technology, Sichuan Agricultural University, Ya’an, Sichuan, China; Tuscia University, Italy

## Abstract

Factors that determine the genetic structure of species in southwestern China remain largely unknown. In this study, phylogeography and demography of *Tylototriton shanjing* was investigated from a mitochondrial perspective to address the role of the Quaternary ice ages in shaping phylogeographic history and genetic diversity of Yunnan. A total of 146 individuals from 19 populations across the entire range of the species were collected. We detected four maternal phylogenetic lineages corresponding to four population groups, and found that major glaciation events during the Pleistocene have triggered the intra-specific divergence. Coalescent simulations indicated that the populations retreated to different refugia located in southern Yunnan, northwestern Yunnan, the border region of western Yunnan with Myanmar, and middle-western Yunnan, respectively, during previous glacial periods in the Pleistocene, and these four refugia were not retained during the Last Glacial Maximum. Population expansions occurred during the last inter-glaciation, during which ice core and pollen data indicated that the temperature and precipitation gradually increased, and declines of population sizes started after the beginning of the Last Glacial Maximum when the climate became cooler and dryer. The paleo-drainage system had no contribution to the current genetic structure and the rivers were not dispersal barriers for this salamander.

## Introduction

Quaternary climatic oscillations had profound effects on the geographical distribution and genetic structure of many species. Population genetic analyses have shown that during the Pleistocene, many floral and faunal species in the Northern Hemisphere migrated southward and survived in refugia during glacial periods and subsequently expanded northward from southern refugia during interglacial periods [1]. China is an important global Pleistocene refugia [2]. The Kunlun-Huanghe Movements (1.1–0.6 Mya) uplifted the Qinghai-Tibetan Plateau to >3000 m a. s. l. and initialized the glaciations in China [3], [4]. Currently, five major glaciations (Xixiabangma, Nyanyaxungla, Zhonglianggan, Guxiang, and the last glaciation) have been recognized to have occurred in the montane regions of western China during the Pleistocene [5], [6], but no continuous ice sheet was present [7] and environmental diversity of the tropics and subtropics in lower elevations was still maintained [8]. Many phylogeographic studies have recently explored the evolutionary consequences of climatic fluctuations and the complex local topography in China. Most of these studies focused on the Qinghai-Tibetan Plateau and its surrounding area (e.g. [9]) and southern (e.g. [10]) and eastern China (e.g. [11]), but less attention has been devoted to southwestern China.

Yunnan is an ideal region to study ecology, distribution and evolution, especially historical geological events, climate oscillations and biological adaptations. Being characterized by dramatic variations in climate and topography, the montane regions of Yunnan support a wide array of habitats. Moreover, during the Quaternary, there was no large-scale continental glaciation in this region [12]. This, combined with a complex, microhabitat-rich topography, would allow species to survive in different refugia and promote intra-specific divergences in small and isolated populations [13].

However, the mechanisms that shape the current genetic structure in Yunnan are still poorly understood, because different species may have different evolutionary trajectories and different response mechanisms to the same historical events. Some studies revealed that lineage divergence times were much earlier than the Pleistocene and genetic structures were shaped by orogenic events [14], [15] or historical drainage rearrangements [16]–[18], and deep river valleys had acted as barriers to gene flow [15], [19]. However, other studies revealed that lineage divergences were triggered mainly by climatic oscillations during the Pleistocene [19]–[21], although past geologic events had also played an occasional role [21]; and historical or contemporary drainage systems did not contribute to current genetic structure [20], [21] (but see [19]). The population structure of a species can be affected by its life history and ecological traits [22], and even sympatric species may have different phylogeographic patterns and genetic structures due to different habitat preferences [21], [23]. Thus, more work is needed to investigate the mechanisms shaping the phylogeographic patterns and genetic structures of species in this region from a comparative phylogeographic perspective.

In addition, an accurate delimitation of glacial refugia is a high priority for conservation because these are key areas for the persistence and evolution of biodiversity [24]. The topographically complex region of Yunnan likely provided stable habitats for the survival of species in different refugia during the ice ages, corresponding to the hypothesis of ‘refugia within refugia’ [25]. A recent study based on plant endemism in China has recognized southern Yunnan as a former glacial refugium [24], and molecular evidence for refugial survival in northwestern Yunnan Plateau has also been recently reported by Zhan et al. [20] and Li et al. [21]. However, up until now only a few studies tried to locate refugia [20], [21]. The sparse literature on this region limits our understanding of the development and sustainment of high diversity within the complicated topography of Yunnan.

Amphibians, being terrestrial poikilotherms with narrow habitat requirements and limited dispersal potentials, are useful model animals for population genetic studies. The range of Red Knobby Newt (*Tylototriton shanjing*) spans the Yunnan province of China, from the tropical south, through the subtropical center and west, to the temperate northwest [26], [27]. It inhabits moist, vegetated areas close to standing water bodies at elevations of 1000–2100 m [28].

In this study, we examine the evolutionary history of *T. shanjing* to test hypotheses involving the following questions: (i) How did climatic changes during the Pleistocene affect the observed phylogeographic structure and demographic dynamics? (ii) Did historical or contemporary drainage systems contribute to the current patterns of genetic variation? Such information will improve our understanding of the historical and ongoing evolutionary driving forces for maintaining the extraordinarily high biodiversity of Yunnan, China.

## Methods

### Ethics Statement

All animal samples were collected following the regulations for the implementation of People’s Republic of China on the protection of terrestrial wildlife (State Council Decree [1992] No. 13) and approved by Wildlife Protection Office, the Forestry Department of Yunnan Province as well as the Ethics Committee of Kunming Institute of Zoology, Chinese Academy of Sciences.

### Sample Collection, DNA Extraction, Amplification and Sequencing

**Figure 1:**
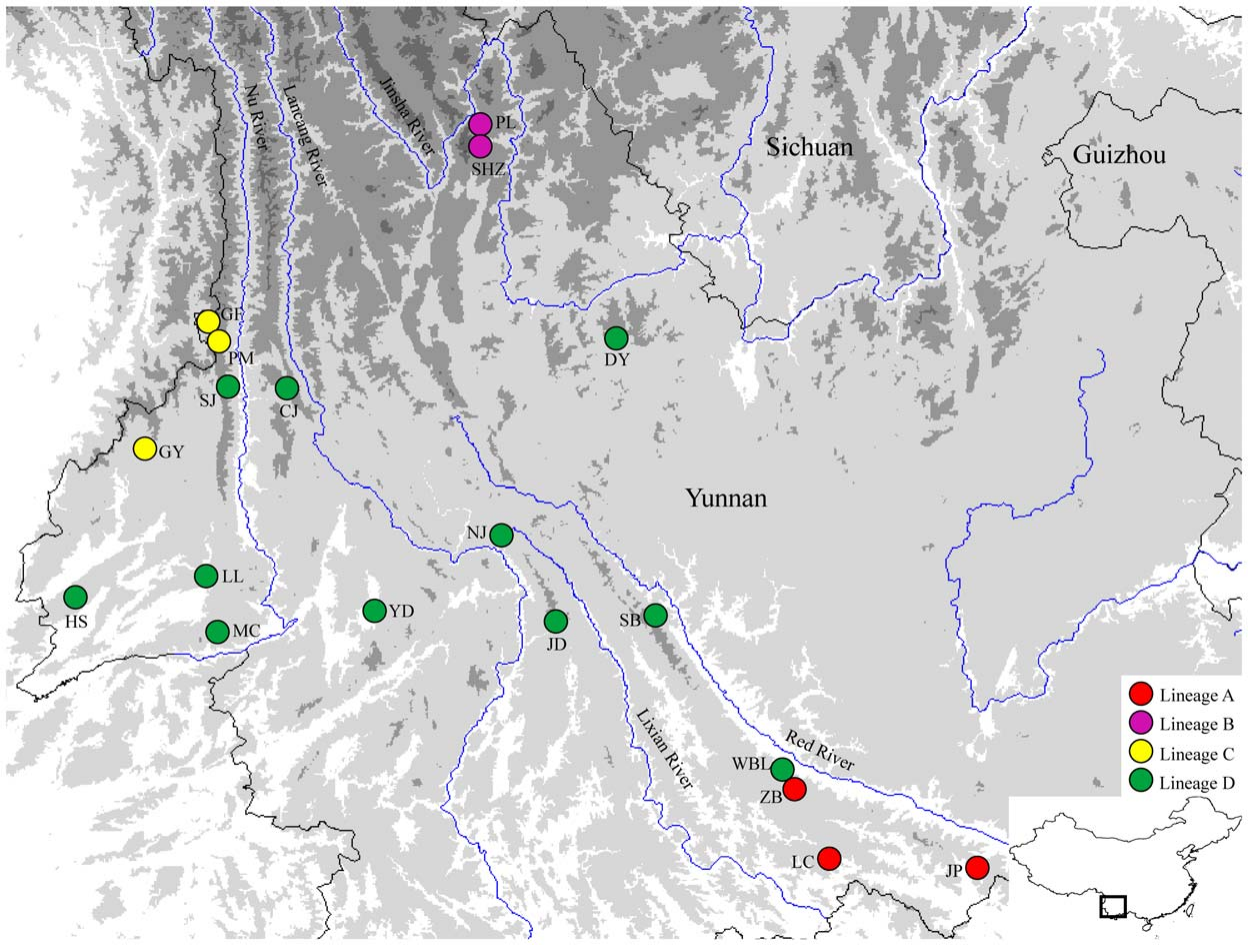
Map showing the sampled populations of *T. shanjing*. Height is reflected by shading and populations are named as in Table 1.

A total of 146 individuals were sampled from 19 populations throughout the entire geographical range of *T. shanjing* (Table 1 and Figure 1). Animals were caught by hand and were sacrificed by diethyl ether anesthesia, and then liver tissues were collected and stored in 99% ethanol immediately after removal. Specimens were fixed in 10% formalin and finally preserved in 70% ethanol. Three congeneric species, *Tylototriton taliangensis*, *Tylototriton kweichowensis*, and *Tylototriton wenxianensis*, were selected as outgroup. Sequences of *T. wenxianensis* were obtained from GenBank (EU880341).

**Table 1:** Sampling localities, phylogroup, sample sizes (*N*), haplotypes and their frequencies, gene diversity, nucleotide diversity, and the probability of being the population of origin of the lineage (MRCA %).

Lineage	Population	Haplotypes present (number of individuals)	*N*	Haplotype diversity	Nucleotidediversity	MRCA %
A	Jinping (JP)	H1 (2), H2 (2), H3 (5), H4 (1)	10	0.7333±0.1199	0.0015±0.0009	35.2
A	Lvchun (LC)	H5 (4), H6 (3), H7 (1), H8 (3), H9 (1)	12	0.8182±0.0703	0.0013±0.0008	63.9
A	Zhangba (ZB)	H10 (1),	1	–	–	1.9
B	Peiliang (PL)	H26 (9), H27 (1)	10	0.2000±0.1541	0.0001±0.0001	43.1
B	Shuanghaizi (SHZ)	H28 (8), H29 (2)	10	0.3556±0.1591	0.0002±0.0002	56.9
C	Gangfang (GF)	H43 (5), H44 (2), H45 (1)	8	0.6071±0.1640	0.0004±0.0004	7.46
C	Pianma (PM)	H43 (7), H45 (5), H46 (2)	14	0.6484±0.0813	0.0004±0.0003	46.07
C	Guyong (GY)	H55 (1)	1	–	–	46.47
D	Wubulu (WBL)	H11 (1), H12 (3)	4	0.5000±0.2652	0.0006±0.0006	16.334
D	Shuangbai (SB)	H13 (10), H14 (2), H15 (1)	13	0.4103±0.1539	0.0002±0.0002	3.03
D	Jingdong (JD)	H13 (7), H16 (1), H17 (1), H18 (1)	10	0.5333±0.1801	0.0020±0.0012	3.314
D	Nanjian (NJ)	H19 (1)	1	–	–	0.352
D	Dayao (DY)	H13 (4), H20 (1), H21 (3), H22 (1), H23 (3), H24 (1), H25 (1)	14	0.8681±0.0594	0.0019±0.0011	15.754
D	Yongde (YD)	H30 (2), H31 (5), H32 (1), H33 (3), H34 (1), H35 (1)	13	0.8205±0.0817	0.0018±0.0011	13.144
D	Caojian (CJ)	H36 (1), H37 (1), H38 (1), H39 (1), H40 (1)	5	1.0000±0.1265	0.0021±0.0014	0.686
D	Shangjiang (SJ)	H41 (3), H42 (1)	4	0.5000±0.2652	0.0008±0.0007	0.078
D	Longling (LL)	H47 (2), H48 (2), H49 (1), H50 (2), H51 (3), H52 (1), H53 (1), H54 (1)	13	0.9231±0.0500	0.0021±0.0012	21.32
D	Mucheng (MC)	H56 (1), H57 (1)	2	1.0000±0.5000	0.0017±0.0019	21.34
D	Husa (HS)	H58 (1)	1	–	–	4.646
Total			146	0.9608±0.0078	0.0097±0.0048	

Genomic DNA was extracted from liver tissue fixed in 99% ethanol. Tissue samples were digested using proteinase K, and subsequently purified following a standard phenol/chloroform isolation and ethanol precipitation. Fragments of cytochrome *b* (cyt *b*), control region (CR), tRNA-Phe, and 12S rRNA genes were amplified and sequenced. The cyt *b* fragment was amplified using primers MVZ15 and MVZ16 [29], and a fragment spanning the entire CR, tRNA-Phe and part of the 12S rRNA genes was amplified using primers PRO (D. Buckley, unpublished data; personal communication) and 12SH1478 [30]. PCR amplifications were performed in 50 µl reactions using the following cycling conditions: an initial denaturing step at 94°C for 5 min; 35 cycles of denaturing at 94°C for 1 min, annealing at 46 (for cyt *b*) or 50°C (for the fragment spanning CR, tRNA-Phe, and 12S rRNA) for 30 s, and one minute extension at 72°C, followed by a final extension step at 72°C for 5 min. PCR products were purified via spin columns. Sequencing of cyt *b* was performed directly using the corresponding PCR primers. For sequencing the fragment spanning CR, tRNA-Phe, and 12S rRNA, the corresponding PCR primers and four internal primers designed by this study were used (Table S1). DNA sequences of both strands were obtained using the BigDye Terminator 3.1 on an ABI PRISM 3730 following the manufacture’s instructions.

### Phylogenetic Analyses

DNA sequences were aligned using SEQUENCE NAVIGATOR (Applied Biosystems) and rechecked by eye. Haplotypes were identified using DnaSP 5.0 [31]. Maximum parsimony (MP) analysis was performed in PAUP* 4.0B10 [32] using a heuristic search with 1000 random-addition sequence replicates and support for nodes of the resulting MP tree was assessed by analyses of 1000 bootstrap replicates. Bayesian inference (BI) analysis was conducted in MrBayes 3.1.2 [33] based on the best-fit substitution model selected by Modeltest 3.7 [34], and two runs were performed simultaneously with four Markov chains starting from random trees. The Markov chains were run for 2×10^6^ generations and sampled every 100 generations. Convergence was confirmed by plots of Ln *L* scores and low standard deviation of split frequencies. The first 2×10^5^ generations were discarded as burn-in, and the remaining trees were used to create a 50% majority-rule consensus tree and to estimate Bayesian posterior probabilities.

### Estimating Divergence Times

Time to the most recent common ancestor (TMRCA) was estimated using an uncorrelated lognormal relaxed molecular clock model as implemented in BEAST 1.6.2 [35]. To calibrate the rate of divergence, a closest external calibration point was incorporated based on the assumption that divergence between *Pleurodeles waltl* from Iberia and *Pleurodeles poireti* from northern Africa was initiated by a vicariance event at the end of the Messinian salinity crisis, approximately 5.3 Mya [36]–[39]. We did no use other known fossil records within Salamandridae as calibration because they are more external than the divergence between *P. waltl* and *P. poireti* based on Vieites et al. [40], which will lead to overestimation of times to divergence according to Ho et al. [41] and Hugall et al. [42]. Four independent runs were conducted for 10^7^ generations using the HKY+I+G model and sampled every 1000 generations, assuming a coalescent prior of constant population size. These four runs were then combined in Tracer 1.4 [43] to visualize the results of each run as well as examine the effective sample size (ESS) for each parameter, following a burn-in of initial 10% cycles. Mean rate was estimated in this analysis.

### Population Analyses

Genetic diversity was estimated by haplotype diversity (h) and nucleotide diversity (π) in Arlequin 3.1 [44]. Three approaches were used to examine geographic partitioning of genetic diversity. First, the haplotype network was estimated using TCS 1.21 [45] based on the 95% parsimony criterion to infer the genealogical relationships among haplotypes. Second, a spatial analysis of molecular variance (SAMOVA) [46] was used to infer population groups. This method uses a simulated annealing procedure to maximize the proportion of total genetic variance due to differences between groups of populations. Third, potential effect of river systems on genetic structure was investigated by analysis of molecular variance (AMOVA) in Arlequin 3.1.

### Analysis of Demographic History

Three commonly used methods were applied to examine demographic history. First, we traced population growth using Tajima’s *D* statistics [47] and Fu’s *F*s test [48] estimated by Arlequin. Significance of *D* and *F*s values were assessed through 1000 simulations. Under demographic expansion, both of these indexes are expected to have significant negative values.

Second, evidence for population expansion was tested by examining pairwise mismatch distributions in Arlequin. Under the null hypothesis of sudden expansion, the raggedness index quantifying the smoothness of the observed mismatch distribution [49] and the sum of squared deviations (SSD) between observed and expected mismatch distribution were computed, and the statistical significance was tested by *P*_Rag_ and *P*_SSD_, respectively, using a bootstrap approach (1000 replicates). The time at which the population expansion began (*t*) can be estimated from the relationship τ = 2*ut* [50], where τ is the mode of the mismatch distribution and *u* is the estimated mutation rate of the sequences.

Third, past population dynamics were examined using Bayesian skyline plots [51], a model-free coalescent-based method implemented in BEAST 1.6.2. The mutation rate was fixed to be the mean substitution rate estimated above. Following the HKY+I+G model, 10^7^ iterations of the MCMC chains were run, sampling every 1000 iterations and discarding the first 10% as burn-in. Other settings of Bayesian prior were default. Bayesian skyline plots were generated with Tracer 1.4.

### Tests of Glacial Refugia Assumptions Based on Coalescent Simulations

**Figure 2:**
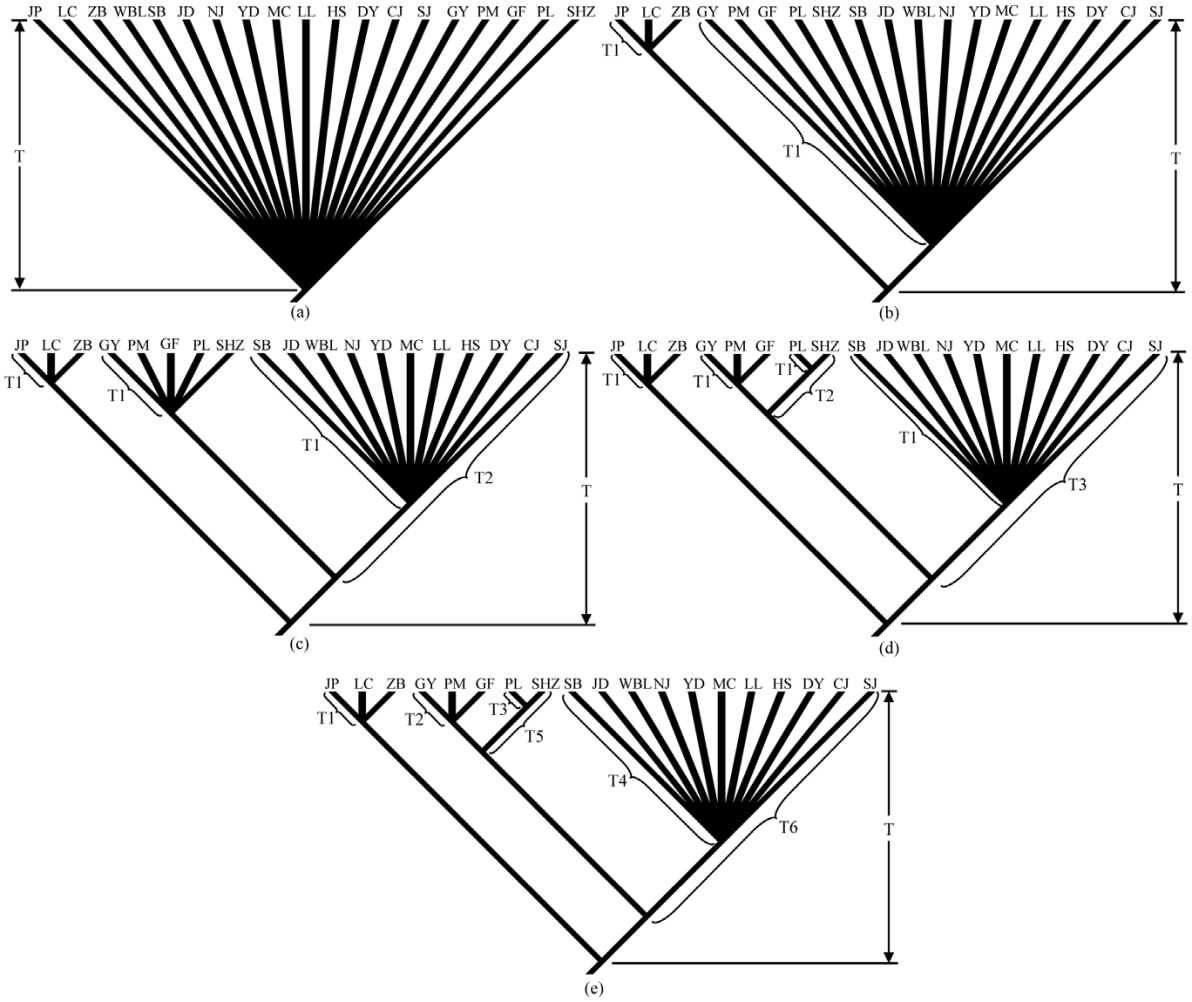
Models of coalescent simulation used to test refugial hypotheses. (a) Hypothesis of single refugium at the end of the Last Glacial Maximum (T = 18 000 years BP); (b) Hypothesis of two refugia at the end of the Last Glacial Maximum (T1 = 18 000 years BP, T = 830 000 years BP); (c) Hypothesis of three refugia at the end of the Last Glacial Maximum (T1 = 18 000 years BP, T2 = 760 000 years BP, T = 830 000 years BP); (d) Hypothesis of four refugia at the end of the Last Glacial Maximum (T1 = 18 000 years BP, T2 = 710 000 years BP, T3 = 760 000 years BP, T = 830 000 years BP); (e) Hypothesis of four refugia before the Last Glacial Maximum (T1 = 250 000 years BP, T2 = 290 000 years BP, T3 = 70 000 years BP, T4 = 530 000 years BP, T5 = 710 000 years BP, T6 = 760 000 years BP, T = 830 000 years BP). The detail interpretation for these models is given in the text.

Coalescent simulations of genealogies constrained within models of population divergence provide a powerful means of assessing the fit of observed genetic patterns to different phylogeographic hypotheses [52]. To test for the hypothesis that there were multiple glacial refugia rather than a single refugium during Quaternary climatic oscillations, 1000 coalescent genealogies were generated in Mesquite 2.74 [53] under the five historical scenarios of Pleistocene refugia (Figure 2), and the distribution of *S*, the minimum number of sorting events required to explain population subdivision [54], was recorded. Then, we evaluated model fit by comparing the *S*-value of the BI genealogy and the *S*-values of the simulated genealogies.

Effective population size (*N*e) for the simulations was estimated using the mutation parameter *θ* (*Theta-W*) calculated by DnaSP. We converted the theta to *N*e using the equation *θ* = 2*N*eμ, with the estimated mean substitution rate. During coalescent simulations, the overall *N*e was set to equal the empirical estimate, and the *N*e of the refugial population was constrained to a size proportional to the overall *N*e. Absolute time (years) was converted to coalescent time (generations), assuming a generation time of 4 years. TMRCA dates estimated from BEAST were used to construct hypotheses of more than one refugium (detailed in Results).

### Refugia Localization

Following Dépraz et al. [55], refugia localization reconstructon (RLR) was used to locate refugia for each major group. From the individual-based Bayesian skyline plot analysis (see above), we randomly selected for each clade 500 genealogies from the post-burnin sample using the program RT.PY [56]. Then we treated the sampling locality as a character of the individual and reconstructed the state of the ‘locality’ character for each node in an unordered parsimonious fashion in Mesquite 2.74.

The number of times that each sampling site was inferred as the unique origin of the root was added over the 500 genealogies. When several *n* localities were equally likely as the common ancestor location, 1/*n* was added to the final score of each sampling site. Then the final sum was divided by the number of genealogies checked (500 here) to give a relative probability score for each locality of having harboured the MRCA of the clade.

## Results

### Sequence Characteristics

The aligned sequences of cyt *b*, CR, tRNA-Phe, and 12S rRNA were 753, 734, 69, and 817 bp in length, respectively. No insertions or deletions were observed for the protein-coding cyt *b* sequences. Among the 146 individuals, a total of 58 haplotypes were identified by 144 polymorphic sites when all fragments were combined. Sequences of these haplotypes and that of outgroup have been deposited in GenBank under accession numbers JX854457–JX854515, JX854517.

### Phylogeny and Divergence Time

**Figure 3:**
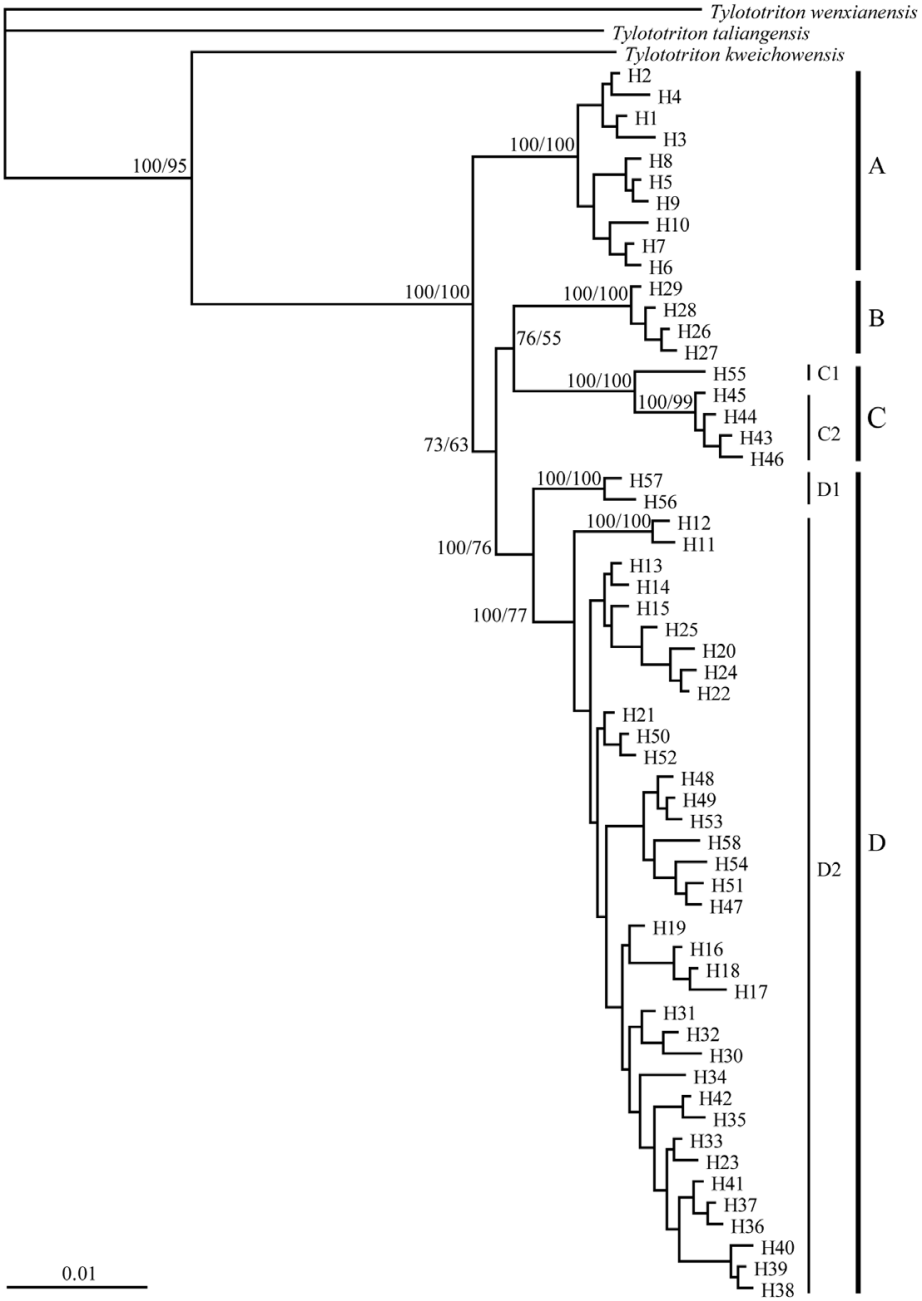
Bayesian inference tree for *T. shanjing* based on the 58 haplotypes from cytochrome *b*, control region, tRNA-Phe, and 12S rRNA sequences. The nodal numbers are BPP and MP bootstrap value, respectively.

The HKY+G+I model was selected as the best-fit model of nucleotide substitution. MP and BI analyses obtained consistent tree topologies (Figure 3). Monophyly of the 58 haplotypes was strongly supported and four major lineages were recognized (A–D), with two of these (C and D) being further subdivided into two sub-lineages (C1 and C2, D1 and D2). Lineage A was restricted to southern Yunnan, lineage B was restricted to northwestern Yunnan, lineage C was restricted to the border region of western Yunnan with Myanmar, and lineage D was comprised of haplotypes from the middle-western Yunnan. In all cases, lineage D was the sister group to the clade of lineages B and C, and A was the sister group to the clade (D, (B, C)).

**Figure 4:**
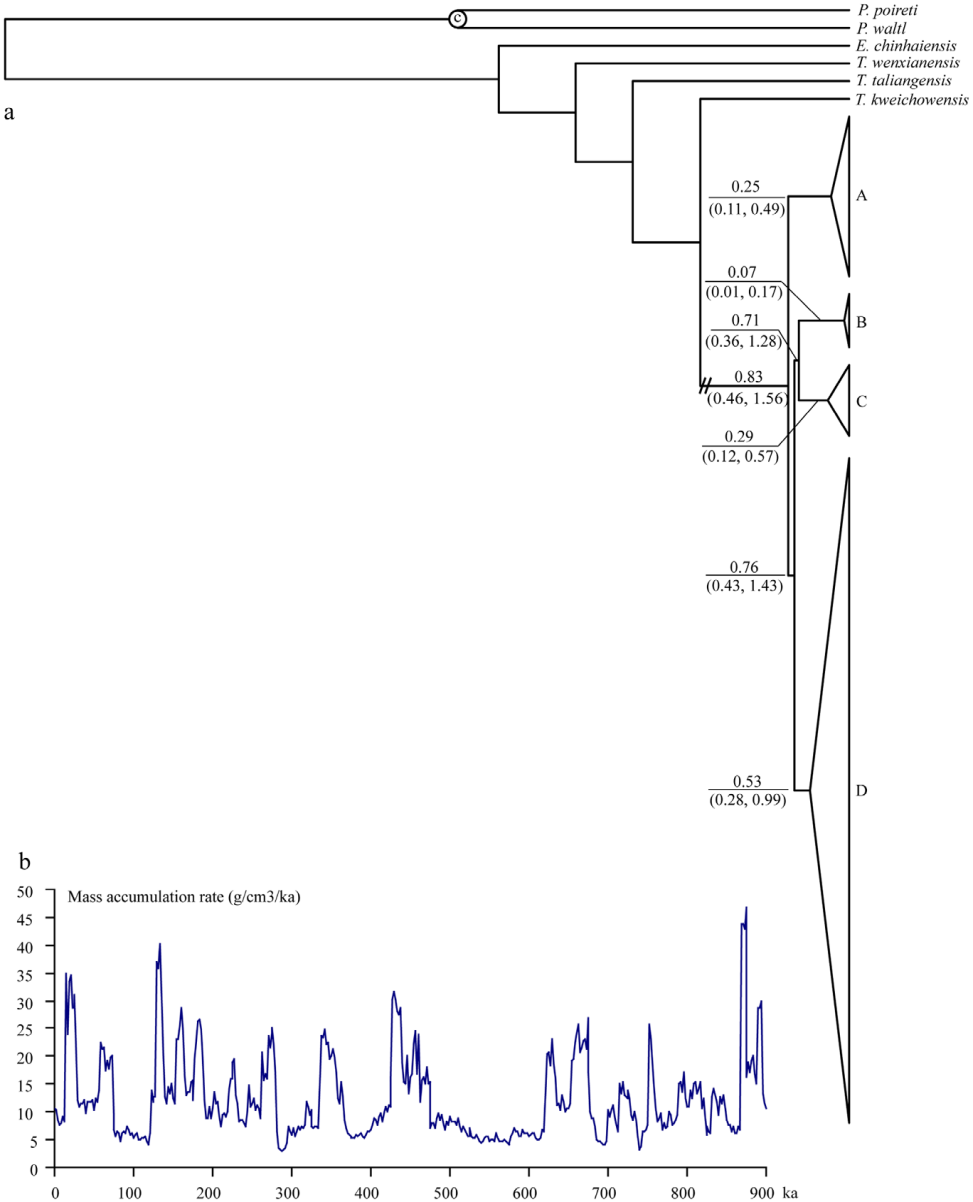
Dated phylogeny and mass accumulation rate of loess. (a) Simplified dated BEAST-derived tree showing divergence dates and TMRCA in Mya with 95% highest probability density (HPD) for major nodes and lineages within *T. shanjing*; (b) Changes in mass accumulation rate of loess deposits on the Chinese Loess Plateau since 0.9 Mya. The data were derived from Sun and An [74].

The divergence time of lineage A from all other lineages was estimated to be approximately 0.83 Mya (95% HPD: 0.46–1.56; Figure 4a). Subsequently, lineage D separated from lineages B and C approximately 0.76 Mya (95% HPD: 0.43–1.43), and lineage B separated from lineage C approximately 0.71 Mya (95% HPD: 0.36–1.28).

### Population Structure

TCS analysis obtained four haplotype networks at 95% probability (connection limit = 21), being consistent with the four major lineages obtained by the phylogenetic analyses (Figure S1). SAMOVA produced similar significant Φ_CT_ values when the numbers of population groups were assumed to be from four to seven (Table 2). Although populations grouped in lineage D are separated by Nu River, Langcang River, Lixian River, and Red River (Figure 1), AMOVA revealed that the values of Φ_CT_ are not significant (*P*>0.05) and genetic variations among population within groups and within populations are obviously higher than variation among groups when they were partitioned into three or five groups according to the river systems (Table 2), indicating that the river systems had no effect on the genetic structure of these populations.

**Table 2:** The results of SAMOVA for grouping of all populations and AMOVA for grouping of populations within lineage D.

Grouping option	Φ_ST_	Φ_SC_	Φ_CT_	Among groups	Among population within groups	Within populations
2 groups [JP, LC, ZB, WBL, SB, JD, NJ, DY, PL, SHZ, YD, CJ, SJ, LL, MC, HS][GF, PM, GY]	0.93097**	0.85584**	0.52115**	52.11%	40.98%	6.9%
3 groups [WBL, SB, JD, NJ, DY, PL, SHZ, YD, CJ, SJ, LL, MC, HS] [JP, LC,ZB] [GF, PM, GY]	0.92294**	0.79623**	0.62184**	62.18%	30.11%	7.71%
4 groups [WBL, SB, JD, NJ, DY, YD, CJ, SJ, LL, MC, HS] [GF, PM, GY][PL, SHZ] [JP, LC, ZB]	0.91787**	0.60235**	0.79346**	79.35%	12.44%	8.21%
5 groups [PL, SHZ] [GF, PM, GY] [JP, LC, ZB] [WBL] [SB, JD, NJ, DY, YD,CJ, SJ, LL, MC, HS]	0.91562**	0.56252**	0.80712**	80.71%	10.85%	8.44%
6 groups [PL, SHZ] [MC] [GF, PM, GY] [WBL] [JP, LC, ZB] [SB, JD, NJ, DY,YD, CJ, SJ, LL, HS]	0.91466**	0.52669**	0.81970**	81.97%	9.50%	8.53%
7 groups [PL, SHZ] [LC, ZB] [WBL] [SB, JD, NJ, DY, YD, CJ, SJ, LL, HS] [JP][MC] [GF, PM, GY]	0.91379**	0.49128**	0.83053**	83.05%	8.33%	8.62%
Lineage D: [WBL, SB, JD, NJ, DY, YD, CJ, SJ, LL, MC, HS]	0.54666**				54.67	45.33
Lineage D: [SJ, LL, MC, HS] [CJ, YD] [JD, NJ, SB, WBL, DY]	0.55219**	0.53275**	0.04161*	4.16	51.06	44.78
Lineage D: [SJ, LL, MC, HS] [CJ, YD] [JD, NJ] [SB, WBL] [DY]	0.53320**	0.65529**	–0.35416*	–35.42	88.74	46.68

* *P*>0.05;

** *P*<0.001.

### Demography

**Figure 5:**
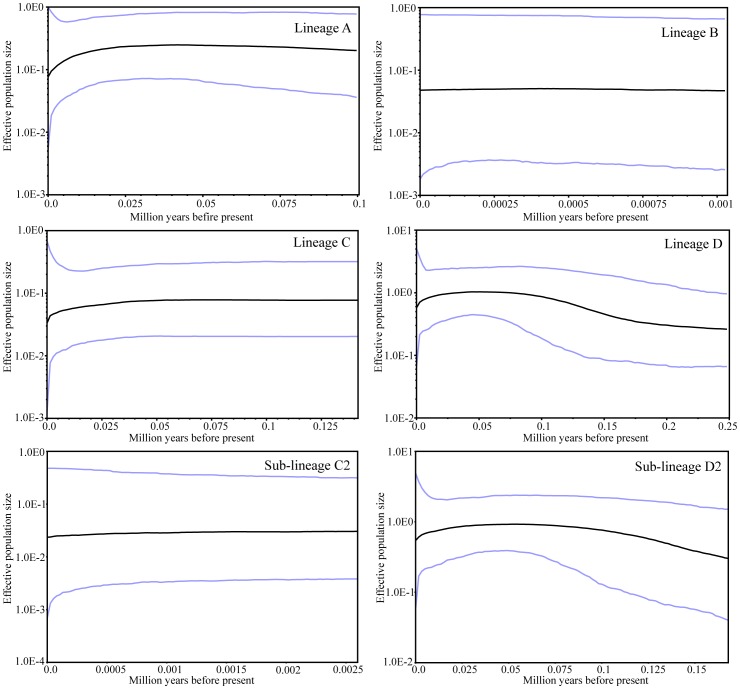
Bayesian skyline plots showing the demographic history of each lineage of *T. shanjing*. Dark lines represent median values for the log_10_ of the population size, and blue lines represent the 95% highest probability density (HPD) intervals.

For lineage D, neutrality tests obtained significantly negative values (*P*<0.05, Table 3) and the mismatch analysis obtained a unimodal curve with non-significant values of SSD and raggedness index (*P*>0.05, Table 3), suggesting that this lineage has undergone population expansions. Considering that lineage D was obviously structured (D1 and D2) and sub-lineage D1 only comprised two individuals from population MC, we further performed analyses only based on sub-lineage D2 and all analyses also supported the hypothesis of expansion. Based on the estimated mean rate of 1.086% substitutions per site per million years, the expansion time of D2 was estimated to be approximately 0.13 Mya, being slightly higher than the estimation from whole lineage of D (Table 3), and the Bayesian skyline plot showed that the *N_ef_* of sub-lineage D2 had a trend towards growth from about 0.175 Mya (Figure 5). For lineages A, B, C, and sub-lineage C2, mismatch analyses did not reject the model of sudden population expansion, but both neutrality tests and Bayesian skyline plots rejected the demographic expansion hypothesis.

**Table 3:** Results of neutrality tests and mismatch analyses.

Lineage	Neutrality tests		Mismatch distribution			
	Fu’s Fs (*P*-value)	Tajima’s *D* (*P*-value)	τ	SSD (*P*_SSD_)	Raggedness (*P*_Rag_)	Expansion time (Mya)
A	0.639 (0.607)	0.287 (0.647)	9.604	0.021 (0.234)	0.035 (0.344)	–
B	–0.472 (0.317)	–0.094 (0.466)	0.979	0.026 (0.099)	0.184 (0.075)	–
C	1.292 (0.776)	–1.948 (0.015)	1.094	0.006 (0.452)	0.048 (0.839)	–
C2	0.021 (0.479)	0.461 (0.712)	1.053	0.001 (0.711)	0.052 (0.821)	
D	–16.513 (0.002)	–1.899 (0.010)	6.156	0.003 (0.940)	0.007 (0.932)	0.119
D2	–15.782 (0.000)	–1.796 (0.013)	6.648	0.003 (0.948)	0.007 (0.937)	0.129

### Glacial Refugia Test

The effective population size was calculated based on the empirical *θ* values and the estimated mean rate (Table S2). The following five hypotheses concerning glacial refugia were tested. First, extant populations were derived from a single refugium at the end of the Last Glacial Maximum (*c*. 0.018 Mya; Figure 2a). Second, the species survived the Last Glacial Maximum in two refugia isolated *c*. 0.83 Mya (Figure 2b). Third, populations from southern Yunnan diverged from populations from other regions *c*. 0.83 Mya, and populations from middle-western Yunnan were isolated from populations from northwestern Yunnan and the border region of western Yunnan *c*. 0.76 Mya, and then extant populations were derived from three refugia at the end of the Last Glacial Maximum (Figure 2c). Both the forth and the fifth hypotheses assumed that populations from southern Yunnan diverged from populations from other regions at the middle Pleistocene (*c*. 0.83 Mya), then populations from middle-western Yunnan became isolated from populations from northwestern Yunnan and the border region of western Yunnan (*c*. 0.76 Mya), with populations from northwestern Yunnan and the border region of western Yunnan diverging into two further lineages (*c.* 0.71 Mya; Figure 2d,e). The difference between the forth and the fifth hypotheses is that the former assumed that extant populations were derived from four refugia at the end of the Last Glacial Maximum (Figure 2d), whereas the later assumed that extant populations were derived from four refugia before the Last Glacial Maximum based on the estimated TMRCA (Figure 2e).

**Figure 6:**
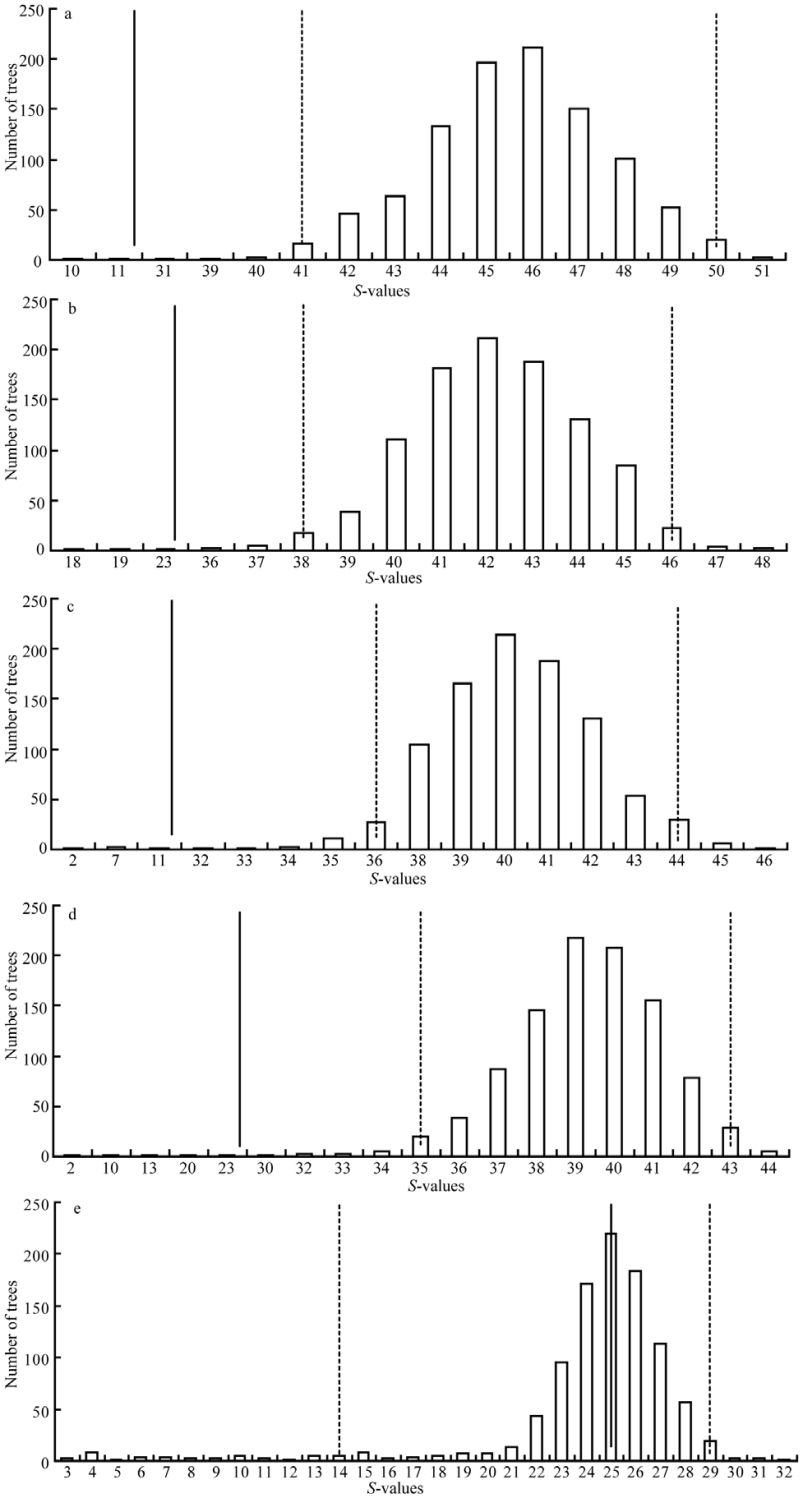
*S*-values for 1000 simulated coalescent genealogies. (a) Results from simulations within the single refugium hypothesis; (b) Results from simulations within the two refugia hypothesis; (c) Results from simulations within the three refugia hypothesis; (d) Results from simulations within the hypothesis of four refugia at the end of the Last Glacial Maximum; (e) Results from simulations within the hypothesis of four refugia before the Last Glacial Maximum. The black line represents the *S*-value for BI genealogy and dashed lines represent the 95% credible interval for the distribution.

We calculated *S* = 25 for our BI genealogy. This value does not fall within the 95% confidence interval of the simulated distribution of *S* for the hypothesis of single refugium, two refugia, three refugia or four refugia at the end of the Last Glacial Maximum but instead falls within the 95% confidence interval of the simulated distribution of *S* for the hypothesis of four refugia before the Last Glacial Maximum (Figure 6). So we reject the hypotheses of a single refugium, two refugia, three refugia and four refugia at the end of the Last Glacial Maximum in favor of the hypothesis of four refugia before the Last Glacial Maximum.

## Discussion

### Effect of Drainage System on Genetic Structure

Our data indicate that *T. shanjing* is comprised of four major maternal lineages and the southern lineage (lineage A) is sister to a clade consisted of the other lineages (Figure 3). The upper Jinsha River drainage originally flowed southward as a tributary to the paleo-Red River [57], under which it could be expected that populations from northwestern Yunnan and the southern Red River drainage area cluster together because they belong to the same paleo-river system. However, in *T. shanjing*, populations from the southern Red River drainage area (lineage A) do not cluster together with populations from northwestern Yunnan (lineage B) (Figure 3). This finding is not compatible with the expectation mentioned above, suggesting that the genetic structure of *T. shanjing* was not shaped by the paleo-river system. Recently, genetic structure of Yunnan pond frog (*Babina pleuraden*) was also found to be not shaped by the paleo-river system [21], but the expected pattern of genetic variation was observed in Yunnan spiny frog (*Nanorana yunnanensis*) [16] and a tree species *Terminalia franchetii* [17], [18]. Variations in ecological adaptations affect geographical patterns of genetic variation [58], [59]. Both *T. shanjing* and *B. pleuraden* live in lentic environments, whereas *N. yunnanensis* and *T. franchetii* are associated with lotic environments. It is likely that the paleo-river system had no impact on the genetic structure of species preferring for standing water because standing water bodies can act as barriers to gene flow among populations [21], [60]. Conversely, the genetic imprint of a paleo-river system can be traced in species associated with lotic environments because flowing water may significantly contribute to the gene flow among populations through the movement of tadpoles and eggs [61].

Although lineage D is distributed across four large rivers (Figure 1), no effect of large rivers could be detected when genetic variation was partitioned by the contemporary river system (Table 2). The species seems to have expanded across these rivers. Populations DY, SB, and JD are separated by the Red River and Lixian River (Figure 1), but H13, the ancestral haplotype of this clade recognized by the TCS analysis, occurs in these three populations (Table 1), suggesting that the Red River and Lixian River are not dispersal barriers for the species. Probably the populations of SB and JD were derived from DY because DY is inferred as one of the origin localities of lineage D (see below) whereas SB and JD have low relative probability of being the population of origin of the lineage (Table 1).

### Lineage Divergence Trigged by Pleistocene Glaciation Events

Geographically no significant east-west barrier exists between the southern lineage (lineage A) and other lineages. The divergence time between the southern lineage and other lineages is estimated to be approximately 0.83 Mya (Figure 4a). This date coincides with a cold stage in the Xixiabangma glaciation (1.17–0.8 Mya; [6]). A high mass accumulation rate for Chinese loess around 0.9 Mya when the glacier expanded extensively [62] supports that it is one of the coldest climatic periods in the region (Figure 4b). Moreover, lineage D diverged from lineages B and C approximately 0.76 Mya, and the timing of the split between lineages B and C is estimated to be approximately 0.71 Mya (Figure 4a). These two divergence events coincide with the Naynayxungla glaciation at 0.78–0.5 Mya, which is the most extensive glaciation occurred during the Middle Pleistocene when there were many large ice caps, glacier complexes and great valley glaciers that covered a total area ≥500,000 km^2^ [6], and another mass accumulation rate peak of Chinese loess (Figure 4b). Following the advances of these glaciations, lineages within *T. shanjing* became restricted to four different regions that served as refugia with interrupted gene flow. With repeated range size changes, surviving populations have passed through genetic isolation, allowing lineages to diverge [13], [59].

The TMRCA of lineages A and C are approximately 0.25 Mya and 0.29 Mya, respectively (Figure 4a). These two dates are consistent with the Guxiang glaciation, the largest glaciation occurred along the southeastern edge of the Tibetan Plateau during Marine Isotope Stages 8–6 [6], and we infer two refugia for *T. shanjing* located in southern Yunnan and the border region of western Yunnan, respectively during this harsh climatic period. Population LC has the highest haplotype diversity among the southern populations and the ancestral haplotype of this group (H5) is present at this site (Table 1), suggesting that this site might have served as the main refugium for lineage A. This is consistent with the analysis of refugia localization, which found that population LC has the highest relative probability of being the population of origin of lineage A (Table 1). For lineage C, the refugium is probably located at PM and GY, which have the highest relative probability of being the populations of origin of this lineage. In addition, the TMRCA of lineages D and B are approximately 0.53 and 0.07 Mya, respectively, being congruent with the Naynayxungla glaciation and the early stage of the last glaciation (0.07–0.01 Mya), respectively. Increased accumulation rates of loess (Figure 4b) supports that the climate became cooler and dryer during these periods. We infer two other refugia for the species during the Naynayxungla glaciation and the early stage of the last glaciation, probably localized to WBL, DY, YD, LL, and MC for lineage D, and SHZ for lineage B (Table 1), although this should be treated with caution because of small sampling size for some populations.

On the basis of our date of the TMRCA for all lineages, it seems that all four refugia were not retained during the Last Glacial Maximum (0.023–0.018 Mya). This is supported by our coalescent simulation, which rejects the hypotheses of a single refugium, two refugia, three refugia, and four refugia at the end of the Last Glacial Maximum in favor of the hypothesis that current populations were derived from four refugia before the Last Glacial Maximum (Figure 6). Coalescent simulations of Zhan et al. [20] and Li et al. [21] also rejected the hypothesis of single refugium during the Last Glacial Maximum in favor of the hypothesis of multiple refugia for the blood pheasant (*Ithaginis cruentus*) and *B. pleuraden*, respectively. Being consistent with that there was no unified ice-sheet covering the whole plateau throughout the Quaternary glaciations [12], which is a precondition to the existence of multiple refugia, more and more evidence indicates that survival in multiple refugia is relatively common for species in the montane regions in southwestern China (e.g. [10], [20], [21], [63], [64]).

### Demographic History

Climatic oscillations during the Pleistocene resulted in several glacial-interglacial cycles, which caused expansions and contractions of habitats [59], [65]. Among the four major population groups, only lineage D possesses a signal of recent growth (Table 3 and Figure 5). The hypothesis of population expansion was also supported for sub-lineage D2 by all analyses. Although SSD and raggedness index did not reject the null model of population expansion for lineages A, B, and C and sub-lineage C2, these statistics are conservative [66]. Bayesian skyline plot revealed an increasing population size in sub-lineage D2 since *c.* 0.175 Mya and it was estimated to have expanded at about 0.13 Mya by Mismatch analysis. This estimation is earlier than the Last Glacial Maximum, being consistent with most recent studies of species from western China [9], [16], [20], [21] but being different from expansion events after the Last Glacial Maximum in many European and North American species [13], [59], reflecting the asynchronicity between glaciations of the montane regions in China and Northern Hemisphere glaciation events [67]. The expansion of sub-lineage D2 is likely to be the consequence of the retreat of the extensive glaciation period (0.5–0.175 Mya; Marine Isotope Stages 12–6) and subsequent warmer and wetter climate during the last inter-glaciation (0.175–0.07 Mya) being equivalent to Marine Isotope Stages 5, as indicated by the decreased mass accumulation rate of loess (Figure 4b). Records of ice core and pollen have indicated a series of warm and humid stages during Marine Isotope Stages 5 [68]. An equivalent expansion time was also estimated in a montane bird *Garrulax elliotii* by Qu et al. [69]. Likely, the sympatric distribution of lineages D and A at Yuanjiang County is the consequence of the expansion of sub-lineage D2. For sub-lineage D1, more samples will be needed to investigate its demographic history because detecting population demographic size changes can be difficult with small sample sizes [70].

On the other hand, the Bayesian skyline plot revealed a recent decreasing trend in population size in lineage A and sub-lineage D2 since *c.* 0.025 Mya (Figure 5), a time corresponding to the beginning of the Last Glacial Maximum when the climate became cooler and the mass accumulation rate reached a peak (Figure 4b), which is consistent with the expectation that profound ecological upheavals during cooler periods would have reduced population sizes. Glacial advances decrease temperatures while increasing aridity. Pollen data have revealed that the average annual temperature at that time was about 6°C lower than today [71], and annual precipitation was about 40% of that at present [72]. These changes may have contributed to the declines in population sizes during the Last Glacial Maximum.

Although Bayesian skyline plot also showed a recent decreasing trend in population size since *c.* 0.025 Mya for whole lineage C, this should be treated with caution because lineage C was obviously subdivided and no obvious recent fluctuation in population size was observed for sub-lineage C2 (Figure 5). Moreover, significant negative value of Tajima’s *D* statistics based on whole lineage C supported population expansion (Table 3), but Bayesian skyline plot did not demonstrate expansion for whole lineage C (Figure 5) and value of Tajima’s *D* is positive for sub-lineage C2 (Table 3), possibly reflecting that population subdivision lowers the power of neutrality tests [73]. So inferences based on a single sub-lineage can better reflect demographic history of lineage C and more samples will be needed to investigate demography of sub-lineage C1.

### Conclusions

In summary, *T. shanjing* comprises four maternal lineages and divergences of these lineages were trigged by glaciation events during the Pleistocene. The paleo-drainage system, which shaped the genetic structures of two sympatric species associated with lotic environment, had no contribution to the current genetic structure of this lentic environment dwelling species, implying that phylogeographic studies on species with different life history and ecological traits are necessary to achieve full understanding of historical and ongoing evolutionary driving forces. There were four glacial refugia for *T. shanjing* during Pleistocene climatic oscillations, some of which (e.g. northwestern Yunnan and southern Yunnan) have also been suggested as glacial refugia for other species, meaning that these regions should be important for consideration when developing sound conservation policies in Yunnan. Population expansion occurred prior to the Last Glacial Maximum while declines of population sizes occurred after the beginning of the Last Glacial Maximum. Considering that this study is based entirely on mitochondrial data, nuclear data will be required to obtain a more comprehensive understanding of phylogeography of *T. shanjing*.

## Supporting Information

Figure S1 **Networks of the 58 haplotypes from cytochrome** ***b*** **, control region, tRNA-Phe, and 12S rRNA sequences of** ***T. shanjing*** **.**
(TIF)

Table S1 **Primers used in this study to amplify and sequence.**
(DOC)

Table S2 **Estimations of the empirical theta values and effective population size.**
(DOC)
