# Supplementary figures and images for: Effect of Pleistocene Climatic Oscillations on the Phylogeography and Demography of Red Knobby Newt (Tylototriton shanjing) from Southwestern China
Source: PLoS One. 2013 Feb 12;8(2):e56066. doi: 10.1371/journal.pone.0056066 (PMC3570421; doi:10.1371/journal.pone.0056066)

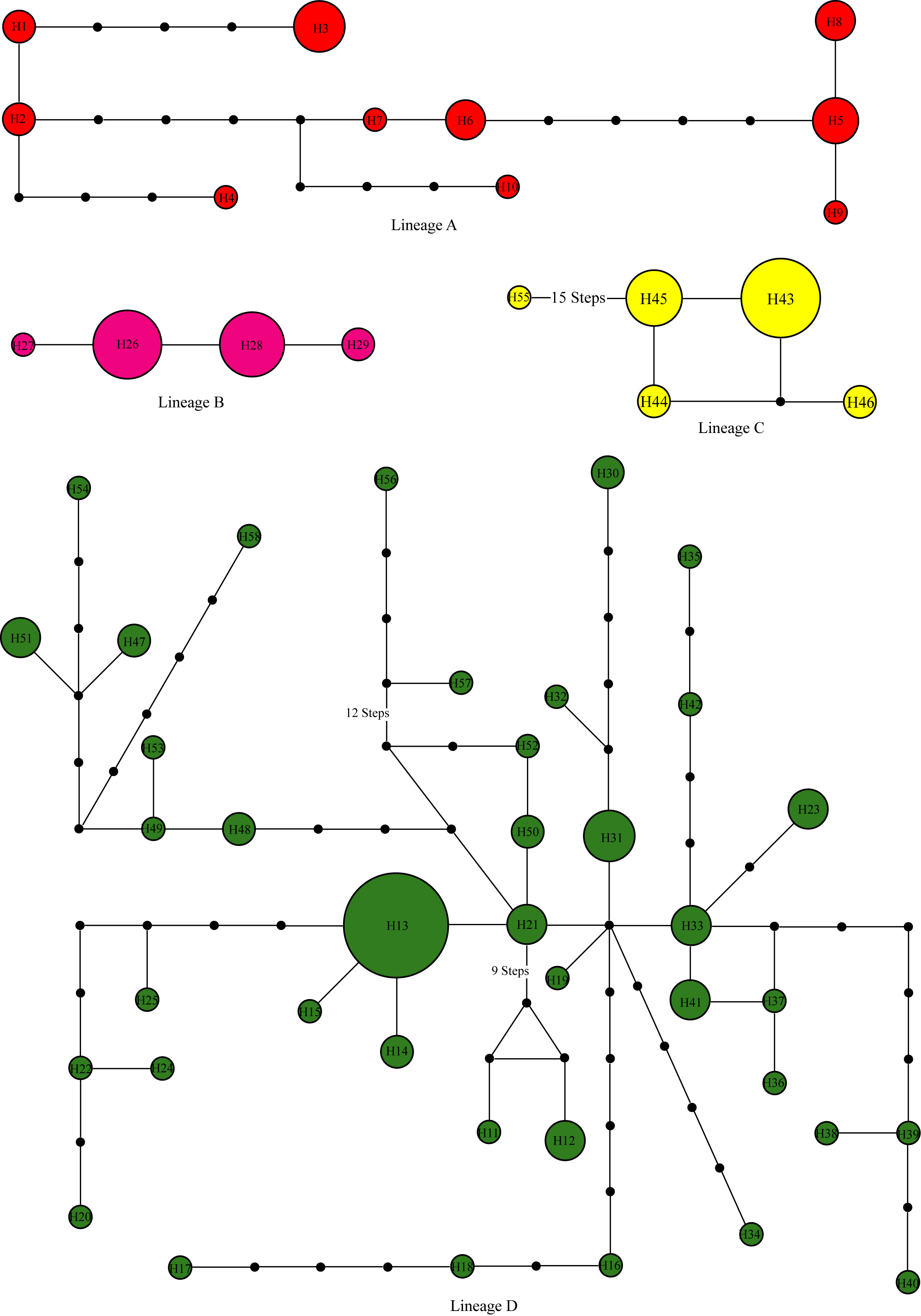

Supplement: Figure S1 — Networks of the 58 haplotypes from cytochrome b , control region, tRNA-Phe, and 12S rRNA sequences of T. shanjing . (TIF) [file pone.0056066.s001.tif]
