# Supplementary material for: Effect of Pleistocene Climatic Oscillations on the Phylogeography and Demography of Red Knobby Newt (Tylototriton shanjing) from Southwestern China
Source: PLoS One. 2013 Feb 12;8(2):e56066. doi: 10.1371/journal.pone.0056066 (PMC3570421; doi:10.1371/journal.pone.0056066)
Supplement: Table S1 — Primers used in this study to amplify and sequence. (DOC) [file pone.0056066.s002.doc]

**Table S1** Primers used in this study to amplify and sequence

| Name | Sequence | Source |
| --- | --- | --- |
| MVZ15 | 5’GAACTAATGGCCCACACWWTACGNAA3’ | Moritz *et al*. (1992) |
| MVZ16 | 5’AAATAGGAARTATCAYTCTGGTTTRAT3’ | Moritz *et al*. (1992) |
| PRO | 5’CGCCACTGGCACCCAAGGCCAAAATTCT 3’ | Buckley (unpublished) |
| 12SH1478 | 5’ TGACTGCAGAGGGTGACGGGCGGTGTGT 3’ | Kocher et al. (1989) |
| D_F1 | 5’ TGGTTGTGAATCTATGAACAA 3’ | This study |
| Ty12S_F1 | 5’ AGCCTTACTATTAATTTTAACTACA 3’ | This study |
| Tydloop_R1 | 5’ TAAGCCTAATGTCGTAAGTATGAAG 3’ | This study |
| Amp_12SR | 5’ TCGATTATAGAACAGGCTCCTCT 3’ | This study |
