# Supplementary material for: Effect of Pleistocene Climatic Oscillations on the Phylogeography and Demography of Red Knobby Newt (Tylototriton shanjing) from Southwestern China
Source: PLoS One. 2013 Feb 12;8(2):e56066. doi: 10.1371/journal.pone.0056066 (PMC3570421; doi:10.1371/journal.pone.0056066)
Supplement: Table S2 — Estimations of the empirical theta values and effective population size. (DOC) [file pone.0056066.s003.doc]

**Table S2** Estimations of the empirical theta values and effective population size

| Lineage | *Theta-W* | *N*e |
| --- | --- | --- |
| Total (ABCD) | 0.01093 | 125806 |
| A | 0.00263 | 30272 |
| B | 0.00036 | 4144 |
| C | 0.00206 | 23711 |
| D | 0.00732 | 84254 |
